# Synthesis and Characterization of Boronate Affinity Three-Dimensionally Ordered Macroporous Materials

**DOI:** 10.3390/polym16111539

**Published:** 2024-05-29

**Authors:** Zhipeng Li, Luxia Zhang, Xiangyu Han, Qinchen An, Mengying Chen, Zichang Song, Linyi Dong, Xianhua Wang, Yang Yu

**Affiliations:** Tianjin Key Laboratory on Technologies Enabling Development of Clinical Therapeutics and Diagnostics, School of Pharmacy, Tianjin Medical University, Tianjin 300070, China; lizhipeng152226@163.com (Z.L.); m15735177825_1@163.com (L.Z.); handream@foxmail.com (X.H.); aqc2001@163.com (Q.A.); chenmengying1999@163.com (M.C.); szc380606848@163.com (Z.S.); donglinyi@tmu.edu.cn (L.D.)

**Keywords:** cis-dihydroxyl, boronbate affinity, 3DOM, colloidal templating method, microspheres

## Abstract

Sample pretreatment is a key step for qualitative and quantitative analysis of trace substances in complex samples. Cis-dihydroxyl (cis-diol) group-containing substances exist widely in biological samples and can be selectively bound by boronate affinity adsorbents. Based on this, in this article, we proposed a simple method for the preparation of novel spherical three-dimensionally ordered macropore (3DOM) materials based on a combination of the boronate affinity technique and colloidal crystal template method. The prepared 3DOM materials were characterized using Fourier transform–infrared spectroscopy, scanning electron microscopy, X-ray photoelectron spectroscopy, and thermo-gravimetric analysis, and results showed that they possessed the characteristics of a high specific surface area, high porosity, and more boronic acid recognition sites. The adsorption performance evaluation results showed that the maximum adsorption capacity of the boron affinity 3DOMs on ovalbumin (OVA) could reach to 438.79 mg/g. Kinetic and isothermal adsorption experiments indicated that the boronate affinity 3DOM material exhibited a high affinity and selectivity towards OVA and adenosine. Sodium dodecyl sulfate–polyacrylamide gel electrophoresis analysis of the proteins in egg whites was conducted and proved that the glycoprotein in the egg whites could be separated and enriched with a good performance. Therefore, a novel boronate affinity 3DOM material a with highly ordered and interconnected pore structure was prepared and could be applied in the separation and enrichment of molecules with cis-diol groups from complex samples with a good selectivity, efficiency, and high throughput.

## 1. Introduction

Sample pretreatment is a key step for qualitative and quantitative analysis of trace substances in complex samples [1]. The objectives of sample pretreatment include increasing the concentration of low-response target molecules, eliminating the interfering substances in the sample matrix, and converting analytes into a state that is easier to detect and separate. The ultimate purpose is to improve the accuracy and sensitivity of analysis. However, due to the complexity of biological samples, specific capture and targeting of certain biomolecules could be rather difficult in many applications.

In recent years, boronate affinity materials have attracted extensive attention due to their unique selectivity and reversible binding with cis-dihydroxyl (cis-diol) group-containing biomolecules including glycoproteins [2,3,4], carbohydrates [5,6,7], nucleosides [8,9], and saccharides [10]. The basic principle of boronate affinity technology is that, under alkaline conditions (pH > pKa), cis-diol groups can bind with boronic acid ligands on adsorbent surfaces to form reversible five-membered or six-membered cyclic esters. When the system changes to an acidic environment, the borate ester bonds break, and the cis-diol-containing compounds can be released [10,11,12]. Due to its high selectivity and convenience in capture and release (pH switch), boronate affinity materials of various kinds, including macroporous monomers [3,13,14,15,16], nanoparticles [4,17,18], and boronic acid affinity-based molecularly imprinted polymers [2,7,11,19], have been prepared. The application of boronate affinity materials has developed rapidly in molecular recognition, clinical diagnosis, cancer cell targeting, and nucleotide isolation [10,20,21,22,23]. Borate ester affinity polymers have the advantages of a simple preparation and wide adaptability. However, the specific surface area of these materials is relatively small, which results in fewer boronic acid recognition sites [24]. Therefore, the adsorption capacity of cis-diol-containing compounds is low.

Three-dimensionally ordered macropore (3DOM) materials are novel molecular sieves with uniform and ordered pores [25]. Compared to traditional porous materials, they possess unique advantages, including a strong periodicity of the pore structure arrangement, a narrow pore size distribution, uniform adjustment within a certain range, and optimal mass transfer [26]. Consequently, 3DOM materials are widely utilized in catalysis, separation, the environment, sensors, and batteries [27,28,29,30,31]. However, until now, most 3DOM materials have been prepared in the form of thin films or block monomers [31,32,33,34], and there has been little research on the preparation of spherical 3DOM materials, which has limited the development of these materials in the field of high-efficiency separation and adsorption.

In the past decade, silica nanoparticles have been widely investigated and utilized under several biomedical circumstances due to their unique properties, including their low toxicity, large surface area, and great biocompatibility [35,36]. However, until now, most of the available silica nanoparticles have been amorphous [37], microporous [38], or mesoporous [35,39,40], and there have rarely been any investigations on 3D-ordered macroporous SiO_2_ nanoparticles.

**Figure 1:**
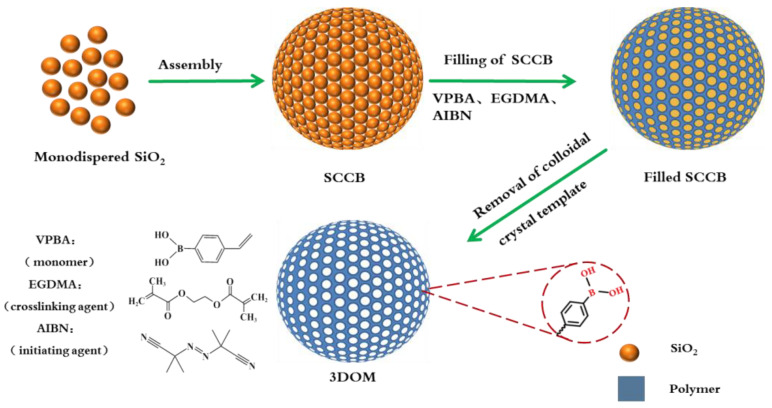
Flow chart for the preparation of boronate affinity 3DOM materials.

Based on these, in this article, we proposed a simple method for the synthesis of boronate affinity 3DOM microspheres with a monodispersed silica (SiO_2_) colloidal template (Figure 1) and applied it to the recognition of compounds containing cis-diol groups. The prepared microspheres had the advantages of a high specific surface area, high porosity, more boronic acid recognition sites, and being suitable for sample pretreatment. They can rapidly separate and enrich cis-diol-containing substances in biological samples with a high throughput.

## 2. Materials and Methods

### 2.1. Materials and Reagents

Dimethyl silicone oil, toluene, cyclohexane, ammonia, acetic acid, and methanol were purchased from Yongda Chemical Reagent Co., Ltd. (Tianjin, China). Methyl methacrylate), hydrofluoric acid (HF, 40% *w*/*v*), horseradish peroxidase, ethylene glycol dimethacrylate (EGDMA), 4-vinyl phenylboronic acid (VPBA), 2, 2′-azobsisobutyronitril (AIBN), and styrene were obtained from Shanghai Macklin Biochemical Technology Co., Ltd. (Shanghai, China). Tetraethyl orthosilicate was obtained from Ailan Chemical Technology Co., Ltd. (Shanghai, China). PEG1000 was obtained from Tianjin Damao Chemical Reagent Factory (Tianjin, China). Ovalbumin (OVA) was obtained from Beijing Kulaibo Technology Co., Ltd. (Beijing, China). Bovine serum albumin (BSA) was obtained from Shanghai Yuanye Biotechnology Co., Ltd. (Shanghai, China). All the other reagents were obtained from Tianjin No. 1 Chemical Reagent Factory (Tianjin, China). All of the reagents were of analytical purity.

### 2.2. Preparation of Silica Colloidal Crystal Template

The silica colloidal crystal template was synthesized through evaporation-induced self-assembly. Monodispersed silica was first prepared using the Stöber method [41] and was introduced as a dispersed phase. Polydimethylsilicone oil was used as the continuous phase. While stirring at 300 rpm, the dispersed phase was slowly dropped into the continuous phase. Due to the surface tension and shear force at the water–oil interface, the SiO_2_ suspension was divided into numerous small droplets containing numerous monodisperse SiO_2_ nanoparticles. Under the condition of 70 °C and stirring, the water in the small droplets slowly evaporated, and SiO_2_ nanoparticles began to aggregate into an ordered silica colloidal crystal ball (SCCB). The assembly was completed when the solution became transparent. The solution was then cooled at room temperature and left overnight to allow the SCCB to precipitate at the bottom of the flask. The obtained SCCB was calcined at 500 °C to improve its mechanical stability and adhesion.

### 2.3. Preparation of Boronate Affinity 3DOM

The filling liquid was prepared using 150 mg of VPBA, 1.44 mL of EGDMA, and 0.02 g of AIBN (initiator) dissolved in 200 μL of DMSO. SCCBs were added to the filling liquid and left for 2 h under a vacuum for complete filling. The excess filling liquid was dumped. Hot-melt polyethylene glycol 1000 (PEG1000) was added, mixed uniformly using a vortex and placed into a refrigerator for 5 min. Finally, the mixture was kept 60 °C for 12 h. After the completion of the reaction, the product was washed with hot water and anhydrous ethanol to remove the PEG1000 and other unreacted impurities. Four percent HF (*w*/*v*) was prepared by diluting 40% HF using distilled water and was added to the above product to remove the SCCB template.

### 2.4. Characterization

The surfaces of the monodispersed silica, SCCB, and boronate affinity 3DOM material were observed and characterized using a scanning electron microscope (SEM, Zeiss, Germany). Fourier transform–infrared (FT-IR) spectra of the monodispersed silica, SCCBs, filled SCCBs, and 3DOM materials were characterized using a Nicolet 380 FT-IR spectrometer (Thermo Fisher, Waltham, MA, USA) using the KBr method. X-ray photoelectron spectroscopy (XPS) of the fabricated microspheres was performed using an ESCALAB™ 250Xi+ X-ray photoelectron spectrometer (Thermo Fisher, Waltham, MA, USA). Thermogravimetric analysis (TGA) of the 3DOM material was performed using a NETZSCH TG 209 F3 thermogravimetric analyzer (Netasch Tarsus, Selb, Germany). The boronate affinity 3DOM material was also characterized based on a pore size analysis using a Pore Distribution Analyzer instrument (Gold APP Instruments Corporation, Beijing, China). The specific surface area was measured using the Brunauer–Emmett–Teller (BET) method.

### 2.5. Adsorption Experiment

In order to quantitatively evaluate the maximum binding amount of OVA to boron-compatible 3DOM materials, a series of OVA solutions with different concentrations (500 ppm–3000 ppm) were prepared, and a standard curve was made for the subsequent concentration calculation using an ultraviolet–visible (UV-Vis) spectrophotometer (Hitachi, Tokyo, Japan) at 280 nm. A total of 5 mg of 3DOM material was added to 2 mL of OVA solution. After the adsorption reaching the equilibrium, the concentrations of the free substrate in the solutions were measured. The adsorption amount was calculated according to the following Formula (1):

(1) $$Q=\frac{(C_{0}-C_{e})V}{m}$$

where *Q* (mg g^−1^) denotes the quantity of adsorbate adsorbed by the boronate affinity 3DOM material. *C_0_* (μg mL^−1^) is the concentration of adsorbate in the initial sample solution. *C_e_* represents the free concentration of the remaining adsorbate after adsorption by the 3DOM material. *V* (mL) is the volume of sample solution, and *m* is the mass of the 3DOM material.

For the adsorption assay of adenosine, a standard curve was made based on different concentrations of adenosine solution using UV-Vis at 260 nm for future calculations. The adsorption of adenosine was investigated using the same procedure as OVA. For the selectivity evaluation, 5 mg of 3DOM was added to 2 mL of OVA solution or BSA solution of different concentrations, and the adsorption experiment was performed as stated above. All the experiments were repeated three times.

### 2.6. Adsorption Kinetics

A certain amount of boronate affinity 3DOM material was dispersed in 1000 ppm OVA solution and placed on a shaking table for adsorption. Samples were taken at regular intervals within 3–180 min, and the concentration of OVA was measured. The adsorption of OVA by the 3DOM material was calculated, and the obtained data were fitted using a pseudo first-order kinetic equation and pseudo second-order kinetic equation to describe the kinetic adsorption process.

### 2.7. Adsorption Ability for Glycoproteins from Eggs

A fresh egg was purchased from the local market. A certain amount of egg white was taken and diluted 100 times in distilled water. The pH was adjusted to 8.6 using a 0.01 M NaOH solution. Five milligrams of the 3DOM material were added to 2 mL of the egg white solution and shaken overnight at room temperature. The 3DOM material with adsorbed glycoprotein was collected using centrifugation and washed 3 times with 0.02 M phosphate buffer and twice with distilled water. Then the 3DOM was treated with 1 mL of 1% acetic acid solution (*v*/*v*) for 2 h to elute the glycoprotein. The supernatant and elute was concentrated to 100 µL. The egg white solution, supernatant, and elute were subjected to sodium dodecyl sulfate–polyacrylamide gel electrophoresis (SDS-PAGE). The proteins were separated using a 5% concentrated gel and a 12% separation gel. After separation, the gel was washed with distilled water and stained with Coomassie brilliant blue R-250 for 3 h. The stained gel was then washed with decolorizing solution until the protein band was clear. The decolorizing solution was prepared by mixing 50 mL of methanol (final concentration of 10%, *v*/*v*), 50 mL of acetic acid (final concentration of 10%, *v*/*v*), and 400 mL of water.

## 3. Results and Discussion

### 3.1. Preparation of Boronate Affinity 3DOM Material

The preparation procedure of the boronate affinity 3DOM material is illustrated in Figure 1. The procedure which mainly composed of three steps, including the package of SiO_2_ into SCCB, the filling of polymer precursor solution, and the removal of the SiO_2_ template.

The uniformity of SiO_2_ particle size is the precondition for preparing SCCB. The closely compacted and highly ordered SCCB can only form when the particle size of SiO_2_ has a narrow variation range. It is generally considered that particles are monodispersed when the polydispersity index (PDI) is less than 0.05. The monodispersed SiO_2_ nanoparticles were characterized using transmission electron microscopy (TEM) and SEM (Figure S1A,B). The particle size distribution is shown in Figure S1C. As shown, the SiO_2_ nanospheres had a good overall morphology, good sphericity, and monodispersity. They were uniform in size, and the average diameter was about 170 nm. The monodisperse SiO_2_ nanoparticles had a tendency to spontaneously arrange in order, which provided favorable conditions for the subsequent preparation of SCCB. They had a narrow particle size distribution range, and the PDI was about 0.023, which met the requirements of subsequent colloidal crystal assembly.

The influence of particle size on the preparation of SCCB was further investigated. SiO_2_ nanoparticles with different particle sizes (170 nm, 200 nm and 255 nm) were applied to prepare the SCCB (Figure S2). As can be seen, SCCB made from SiO_2_ nanoparticles of 170 nm had a good sphericity. However, those that made from 200 nm and 255 nm SiO_2_ nanoparticles were not spherical and could not be used for further 3DOM material synthesis. Therefore, SiO_2_ nanoparticles of 170 nm were used for further investigation.

### 3.2. Characterization of SCCB and Boronate Affinity 3DOM Material

#### 3.2.1. Morphology Characterization

**Figure 2:**
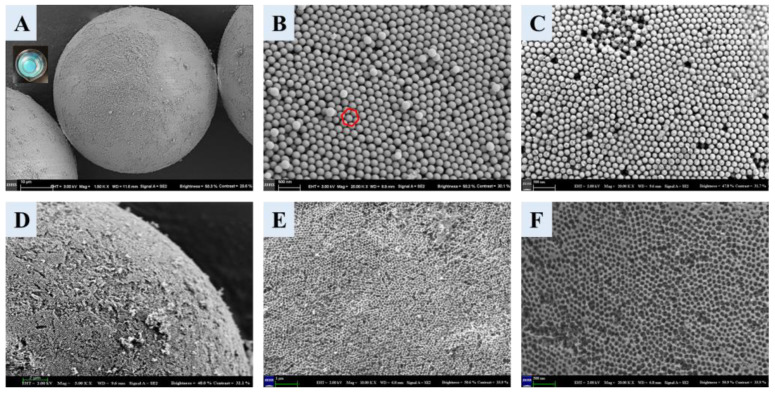
SEM images for whole SCCB (**A**), microscopic structure of SCCB surface (**B**), microscopic structure for surface of filled SCCB (**C**), and SEM images for 3DOM material surface structure (**D**–**F**). Magnification for each image: (**A**), 1500; (**B**), 20,000; (**C**), 20,000; (**D**), 5000; (**E**), 10,000; (**F**), 20,000. The red circle suggested that the surface of the microsphere was a face center cubic (FCC) structure.

The morphology of the materials related to the preparation of the boronate affinity 3DOM material was characterized using SEM. The SCCB was blue when dispersed in ethanol (Figure 2A), which was the structural color [42] produced by the photonic band gap structure of the colloidal crystal, demonstrating the successful synthesis of SCCB. The micro-structure of the SCCB surface is shown in Figure 2B. As we can see, the SCCB was a highly ordered particle that was assembled from numerous closely arranged monodispersed SiO_2_ nanospheres. The surface of the microsphere showed a face center cubic (FCC) structure (red circle in Figure 2B), which proved the successful preparation of the SCCB.The SEM image of the SCCB at a lower magnification is shown in Figure S3. Figure 2C shows the SEM picture of the SCCB filled by the precursor solution, which shows the formation of the filled polymer on the surface and inside of the SCCB. The SEM images of the 3DOM materials after the removal of the template are shown in Figure 2D–F. In the enlarged Figure 2D, an obvious three-dimensional-ordered porous structure can be observed. This structure not only contained a large specific surface area but also provided large pores that were interconnected and easy for mass transferring. The diameter of the macropores in the figure was about 160 nm, which was consistent with the size of the monodispersed SiO_2_ mentioned above, suggesting that the prepared 3DOM completely duplicated the silica colloidal crystal template with the FCC structure. In conclusion, boronate affinity 3DOM materials were synthesized with a highly ordered macropore structure.

#### 3.2.2. FT-IR Spectrum

**Figure 3:**
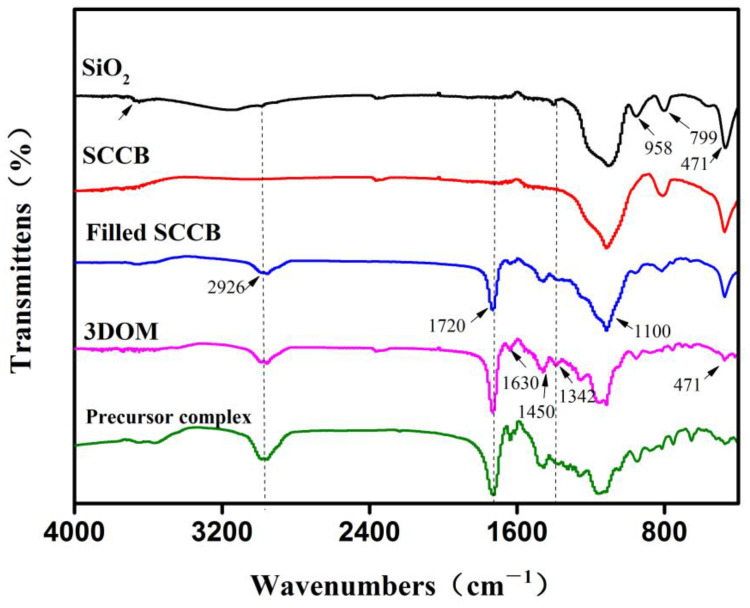
FT-IR images of the materials involved in the preparation of boronate affinity 3DOM material. Important peak changes are labeled with dashed lines.

To further characterize the boronate affinity 3DOM material, an IR spectrum was obtained for each preparation step (Figure 3), which was obtained using the KBr method. In the spectrum for the monodispersed SiO_2_, the absorption peak that appeared at 3500–3700 cm^−1^ was the stretching vibration peak of Si-OH. The broad and strong peak at 1100 cm^−1^ was the asymmetric stretching vibration peak of Si-O bond. The absorption peak that appeared at 958 cm^−1^ belonged to the bending vibration absorption peak of Si-OH. The two absorption peaks at 799 cm^−1^ and 471 cm^−1^ were the symmetric stretching vibration peak and bending vibration peak of the Si-O bond, respectively. In the spectrum for calcinated SCCB, the absorption peaks for Si-OH at 3500–3700 cm^−1^ and 958 cm^−1^ disappeared, suggesting that the Si-OH group was completely condensed into a Si-O-Si bond during the calcination. In the spectrum for the SCCB filled with the precursor solution, compared with the SCCB without filling, the newly appeared absorption peak at 1720 cm^−1^ was the ester carbonyl peak of EGDMA. The absorption peak at 2926 cm^−1^ was the stretching vibration peak of the C-H bond. The two absorption peaks at 1630 cm^−1^ and 1450 cm^−1^ belonged to the benzene ring skeleton vibration peak of VPBA, and the peak at 1342 cm^−1^ was the B-O bond absorption peak from VPBA. The appearance of these peaks demonstrated that the precursor solution had been successfully filled into the SCCB and that polymerization occurred. In the spectrum of the 3DOM material with the template removed, the absorption peak of the silicon oxygen bond at 471 cm^−1^ was significantly decreased compared to the spectra for SiO_2_ and filled SCCB. Moreover, the absorption peak at 799 cm^−1^ disappeared, and the peak at 1100 cm^−1^ was altered. All these changes proved the successful removal of the silica colloidal crystal template by HF. To further prove the removal of the template, the IR spectrum for the precursor solution bulk polymerization product was obtained. As can be seen, the absorption peaks in the spectra for the 3DOM material and precursor complex were consistent, which proved the successful removal of the template.

#### 3.2.3. Thermogravimetric Analysis

**Figure 4:**
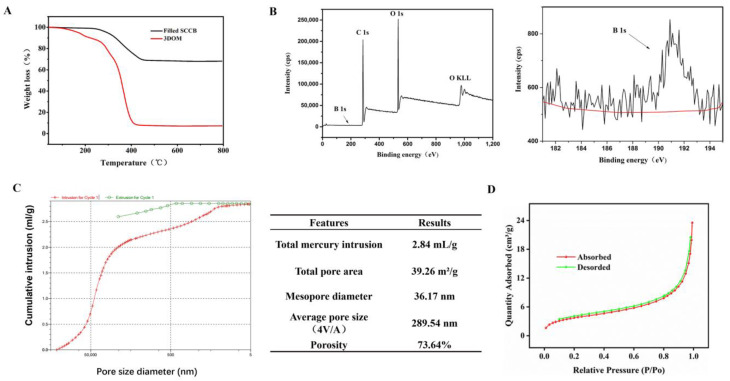
Characterization results for boronate affinity 3DOM materials. (**A**) Thermogravimetric analysis of 3DOM materials. (**B**) XPS spectrum for 3DOM materials. The right part is a magnification of Boron element and the red line showed the baseline of B. (**C**) Pore structure analysis for 3DOM materials. (**D**) BET adsorption and desorption curve.

A thermogravimetric analysis (TGA) was performed using filled the SCCB and 3DOM material at 40~800 °C (Figure 4A). The black curve shows the thermogravimetric properties of the filled SCCB. When the temperature rose from 40 °C to 450 °C, about 30% of the material was lost due to the water evaporation on the material surface and the loss of the filled polymer material. The rest was the silica colloidal crystal template. The red curve was for the boronate affinity 3DOM material. The 3DOM material had almost no residue after calcination at 800 °C, suggesting that there was almost no SiO_2_. Compared with the black curve, it confirmed that the silica colloidal crystal template in the boronate affinity 3DOM material was completely removed.

#### 3.2.4. X-ray Photoelectron Spectroscopy

To verify whether the precursor solution was successfully filled into the voids of the SCCBs, X-ray photoelectron spectroscopy (XPS) was performed for the template-removed 3DOM material (Figure 4B). In the figure, we can see peaks for B 1s (191.01 EV), C 1s (284.82 EV), O 1s (532.71 EV) and other peaks, suggesting that the final 3DOM material contained boron, carbon, oxygen, and other elements. These elements obviously did not exist in the silica template but were from precursor polymers, indicating that the precursor polymers were successfully filled into the voids of the SCCBs.

#### 3.2.5. Pore Structure Analysis

The pore size of the 3DOM material was analyzed using the mercury intrusion method, and the results are shown in Figure 4C. The average pore diameter (4V/A) of the material was 289.54 nm. The porosity was 73.64%, and the diameter of the pore wall was 36.17 nm.

#### 3.2.6. Specific Surface Area

The specific surface area (SSA) of the 3DOM material was measured via a Brunauer–Emmett–Teller (BET) analysis of volumetric nitrogen adsorption isotherms (Figure 4D). The BET specific surface area of the 3DOM material was 13.6592 m^2^/g.

### 3.3. The Effecst of pH on the Adsorption Properties of the Boronate Affinity 3DOM Material

**Figure 5:**
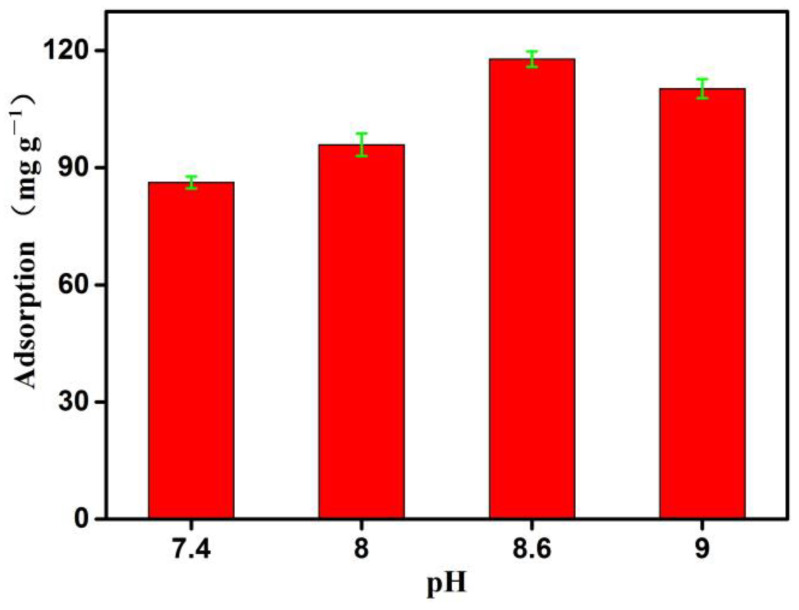
The effects of pH on the adsorption properties of the boronate affinity 3DOM material. The adsorption was measured at room temperature (298.15 K). Data are presented for at least three independent experiments and mean ± SD was shown with green bar for each group.

The adsorption properties of the boronate affinity 3DOM material were evaluated based on its capture of cis-diol groups. The pH of the solution is an important factor affecting the adsorption properties of a material [43], and it is especially important for the reversible covalent reaction between cis-diol-containing compounds and boronic acid ligands [24,44]. Therefore, the adsorption capacity of OVA was evaluated using solutions with different pH values. The pH of the solution was adjusted using a 0.01 M NaOH solution. When the pH increased from 7.4 to 8.6, the adsorption capacity increased significantly from 87.24 mg/g to 117.83 mg/g, reaching a maximum (Figure 5), which was consistent with the pKa (8.62) of 4-vinyl phenyl boronic acid reported in the literature [45]. According to the principle of boronate affinity, under the conditions of low pH (pH < pka = 8.60), only a small amount of inactive boronic acid groups (triangular configuration, SP^2^) can be converted into reaction forms (tetrahedral configuration, SP^3^). Therefore, the adsorption is relatively small at pH7.4 and pH8.0. Although the adsorption is also relatively large at pH9.0, considering the impact of an alkaline environment on OVA proteins [46], the subsequent experiment was carried out at pH8.6.

### 3.4. The Adsorption of OVA by the Boronate Affinity 3DOM Material

#### 3.4.1. Adsorption Dynamics

Adsorption isotherms can provide valuable information about surface properties and dynamic adsorption behavior [47,48]. To evaluate the maximum binding amount of OVA to boron-compatible 3DOM materials, a series of OVA solutions with different concentrations (500 ppm–3000 ppm) were prepared to determine the absorption isotherms. The adsorption amount of OVA was calculated, and the data were fitted using the Langmuir model and Freundlich model, respectively (for formulations, see the Supplementary Information).

**Figure 6:**
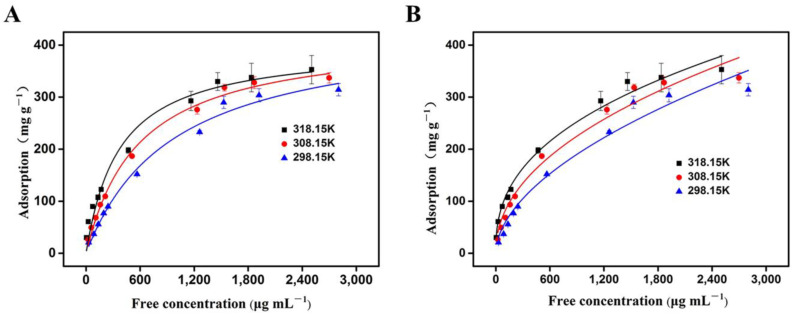
The adsorption of OVA by the 3DOM material fitted with the Langmuir model (**A**) and Freundlich model (**B**). A total of 5 mg of 3DOM was added into 2 mL of OVA solution of different concentrations at a pH of 8.6, and the adsorption was measured at temperatures of 318.15 K, 308.15 K, and 298.15 K, respectively.

As shown in Figure 6, with the increase in the initial concentration of OVA, the adsorption amount gradually increased and finally stabilized (Figure 6A,B). Both models fitted the adsorption data well, since the R^2^ value was close to 1.0 (Table 1). At 25 °C, the R^2^ value of the Langmuir model (0.989) was higher than that of the Freundlich model (0.967), suggesting that the Langmuir equation could better fit the adsorption process. Generally speaking, the Freundlich isotherm model represents adsorption on multi-layer and energy-heterogeneous surfaces and active sites [43], while the Langmuir isotherm model signifies the monolayer adsorption that occurs at a uniform position [45]. Considering that the activated phenylborate group [-B (OH)_3_^−^] (SP^3^) in VPBA can interact with a typical cis-diol unit (from OVA) to form a stable five-membered or six-membered boron ester ring, as well as the existence of macropores in the adsorption material and the uniform distribution of the adsorption site [-B(OH)_2_ group (SP^2^)], we can reasonably draw the conclusion that the Langmuir model can better explain the experimental data. Therefore, it can be considered that the adsorption of OVA by the 3DOM materials occurs through monolayer adsorption. In addition, according to the Langmuir isotherm model, the predicted maximum single-layer OVA adsorption by the 3DOM material at 25 °C was 438.79 mg/g, which was higher than another adsorbent (190.79 mg/g) reported previously [46]. This adsorption ability was higher than or similar to most recently reported boronate affinity adsorbents. For example, the boronic acid-functionalized spherical polymer brushes reported by Chen et al. had a capacity for OVA adsorption of 377.0 mg/g [49]. The boronic acid-functionalized magnetic graphene oxide composite (Fe_3_O_4_-GO@PAAPBA) reported by Su et al. had an adsorption capacity at 471 mg/g for OVA [50]. These results further proved the superior prospect of the 3DOM material for the separation and enrichment of cis-diol compounds, which presented a foundation for future practical applications.

In order to understand the adsorption process from the aspect of energy, the adsorption assay was also carried out at different temperatures. The thermodynamic parameters, the including Gibbs free energy change (G^0^), enthalpy change (H^0^), and entropy change (S^0^) [49], was used to describe the thermodynamic behavior of OVA adsorption by the 3DOM material (Supplementary Information).

As shown in Table 2, at different temperatures, the ΔG^0^ values were all negative, indicating that the adsorption of OVA by the 3DOM material was favorable and feasible. During the adsorption process, ΔG^0^ decreased with increasing temperature, suggesting that the adsorption was spontaneous, and the spontaneity increased with increasing temperatures. The negative value of ΔH^0^ (−37.77 kJ/mol) proved that the adsorption process was exothermic within the experimental temperature range. The positive value of ΔS^0^ suggested that the disorder degree of the solid–liquid interface increased during the adsorption, and the boronate affinity 3DOM material had a high affinity for OVA.

#### 3.4.2. Adsorption Kinetics

In the adsorption kinetics study, the speed and influencing factors for the OVA adsorption by the 3DOM material was investigated. A pseudo first-order kinetic Equation (S5) and pseudo second-order kinetic equation (S6) were applied to describe the kinetic adsorption process (for formulations, see Supplementary Information) [46,51,52].

**Figure 7:**
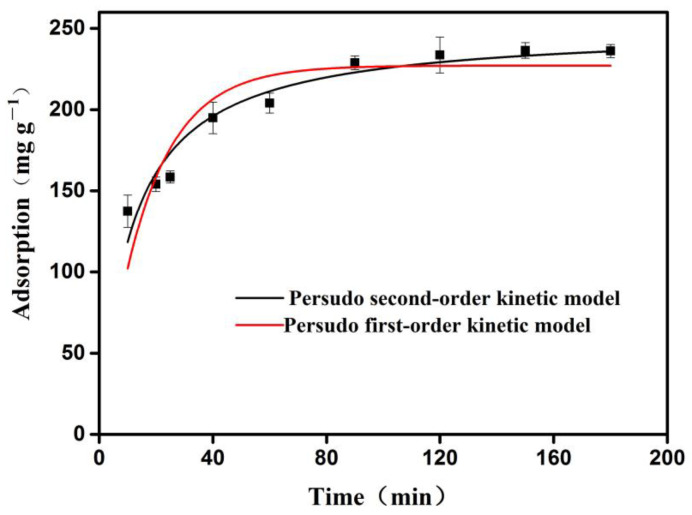
Kinetic fitting of 3DOM material for OVA adsorption. The adsorption was measured at room temperature (298.15 K).

As shown in Figure 7, the red curve is the pseudo first-order dynamic model, and the black curve is the pseudo second-order dynamic model. The adsorption of OVA by the 3DOM material showed typical adsorption kinetic characteristics. As the time increased, the adsorption rate gradually decreased. The reason was that the OVA molecules were adsorbed on the surface of the 3DOM material at the initial stage while encountering resistance after being diffused into the pores. This rapid and efficient adsorption can be attributed to the high porosity and the uniform distribution of the abundant active sites on the 3DOM materials, which was consistent with the above isothermal adsorption analysis results.

The fitted parameters are shown in Table 3. As we can see, the R^2^ value of the pseudo second-order fitting of OVA was 0.932, which was greater than that of the pseudo first-order model (R^2^ = 0.797), indicating that the pseudo-second order model could better describe the adsorption behavior of OVA by the 3DOM material. These data demonstrated that the adsorption of OVA onto the 3DOM materials was based on chemical adsorption, and they further confirmed that there was a strong chemical bond force (reversible covalent bond) between the boronic acid functional group of the 3DOM material and the cis-diol structure of the OVA.

### 3.5. The Adsorption of Adenosine by the Boronate Affinity 3DOM Material

**Figure 8:**
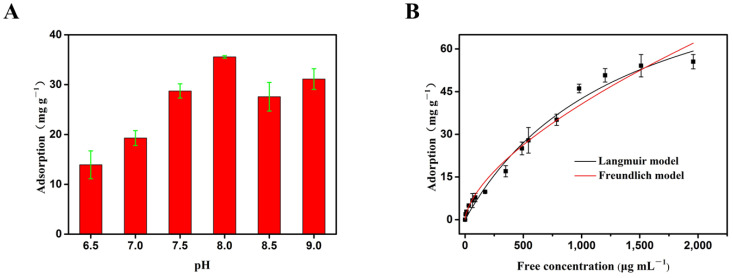
The adsorption of adenosine by the 3DOM material. (**A**) The effects of pH on the adsorption. Data are presented for at least three independent experiments and mean ± SD was shown with green bar for each group. (**B**) The adsorption curve fitted with the Langmuir model and Freundlich model. The adsorption was measured at room temperature (298.15 K).

Following OVA, we measured the adsorption of adenosine, another cis-diol-containing molecule by the boronate affinity 3DOM material. As shown in Figure 8A, the adsorption capacity increased significantly from 13.92 mg/g at pH 6.5 to 38.58 mg/g at pH8.0, where it reached a maximum. Therefore, pH8.0 was chosen for the subsequent experiment.

In order to quantitatively evaluate the maximum binding amount of adenosine by the boronate affinity 3DOM materials, a series of adenosine solutions with different concentrations were used for static adsorption. The data obtained were fitted with Langmuir model and Freundlich model, respectively (Figure 8B and Table 4). With the increase in concentration, the adsorption capacity increased gradually. Both models fitted the adsorption data well, with R^2^ values close to 1.0. Since the R^2^ value of the Langmuir model (0.984) was higher than that of the Freundlich model (0.975), the Langmuir equation could better fit the adsorption process of adenosine. Considering the existence of macropores in the adsorption materials and the uniform distribution of the adsorption site -B(OH)_2_ group (SP^2^), it can be reasonably concluded that the Langmuir model can better explain the experimental data, and the adsorption of adenosine by the 3DOM materials occurred via monolayer adsorption. In addition, according to the Langmuir model, the predicted maximum monolayer adsorption capacity of the boronate affinity 3DOM material for adenosine was 100.28 mg/g, which was higher than the amount of adenosine adsorbed by other previously reported adsorbents [53]. These results further proved that the boronate affinity 3DOM material can be applied to separate and enriched small molecules containing cis-diol groups, with good prospects for the application of small-molecule proteins.

### 3.6. The Selectivity of the Boronate Affinity 3DOM Material

**Figure 9:**
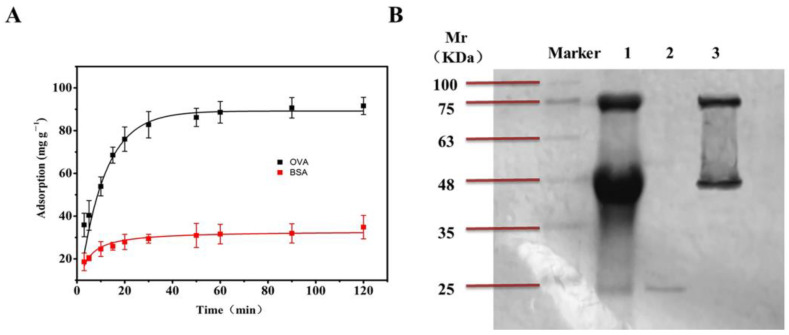
The selectivity and application of BA-3DOM. (**A**) The adsorption of OVA and BSA by the boronate affinity 3DOM material. The adsorption was measured at room temperature (298.15 K). (**B**) SDS-PAGE gel image for protein bands in egg white samples. Lane 1: diluted egg white; lane 2: supernatant after absorption of the 3DOM material; lane 3: eluent of 3DOM material by a 1% acetic acid solution (*v*/*v*).

In order to evaluate the adsorption specificity of the boronate affinity 3DOM materials towards cis-diol-containing compounds, glycoprotein OVA and non-glycoprotein BSA were subjected to adsorption experiments under the same conditions. As shown in Figure 9A, with the increase in concentration, the adsorption of OVA gradually increased and finally stabilized to 314.28 mg/g, while for BSA, the maximum adsorption was only 127.27 mg/g. The reason is that as a non-glycoprotein, BSA contains no cis-diol group and therefore cannot bind to the boronic acid site on the material. The small amount of BSA adsorption was due to non-specific adsorption. In the contrast, OVA contains a cis-diol group that can form a covalent bond with the boronate acid group on the surface of the 3DOM material. Overall, these data indicated that the boronate affinity 3DOM material can selectively adsorb compounds containing cis-diol groups.

### 3.7. Application: Separation and Enrichment of Glycoproteins from Eggs by Boronate Affinity 3DOM Material

To investigate whether the boronate affinity 3DOM material was able to capture cis-diol-containing biomolecules in the presence of other materials and proteins, egg whites from fresh eggs were subjected to the adsorption experiment described above. The proteins were separated using SDS-PAGE, and an image of the stained gel is shown in Figure 9B. Strip1 contained 100 times diluted egg white. Strip2 contained the supernatant after adsorption of the 3DOM material. Strip 3 contained the eluent of the 3DOM material. In egg white samples, glycoprotein OVA (46 kDa), ovotransferrin (76 kDa), and non-glycoprotein lysozyme (17.6 kDa) were obviously observed, but almost no OVA or ovotransferrin were detected in the supernatant (Strip2). Only non-glycoprotein lysozyme was detected in the supernant. After washing, OVA and ovotransferrin were detected in the elute of the 3DOM material (Strip3). These data indicated that the boronate affinity 3DOM was able to capture glycoproteins in the presence of non-glycoproteins.

## 4. Conclusions

In this article, a novel boronate affinity 3DOM material with a highly ordered and interconnected pore structure was prepared and characterized. Moreover, its binding ability to cis-diol-containing molecules was evaluated. The results showed that this material had the advantages of an acceptable specific surface area, high porosity, and more boronic acid recognition sites. As an adsorbent of the glycoproteins in egg whites, the boronate affinity 3DOM material can effectively capture cis-diol-containing glycoproteins in the presence of non-glycoproteins. Therefore, it can be applied in the separation and enrichment of molecules with cis-diol groups from complex samples with a good selectivity, efficiency, and high throughput.

## Figures and Tables

**Table 1:** Fitting parameters of the adsorption equilibrium model of the 3DOM material for OVA.

	Langmuir Model			Freundlich Model		
T(K)	Q_lmax_ (mg/g)	KL (mL/μg)	R^2^	KF (mg/g) (mL/μg) 1/n	1/n	R^2^
318.15	399.72	2.71	0.973	17.05	0.39	0.987
308.15	419.53	1.72	0.994	9.34	0.47	0.972
289.15	438.79	1.04	0.989	4.80	0.54	0.967

**Table 2:** Thermodynamic parameters for the adsorption process of OVA by the 3DOM material.

	$∆H^{0}$ (KJ/mol)	$∆G^{0}$ (KJ/mol)			$∆S^{0}$ [J/(mol·K)]
		298.15 K	308.15 K	318.15 K	
OVA	−37.77	−43.56	−43.73	−43.95	19.38

**Table 3:** Adsorption kinetic parameters of 3DOM material for OVA.

	Pseudo First-Order Kinetic Model			Pseudo Second-Order Kinetic Model		
	Q_e_ (mg g^−1^)	k_1_ (min^−1^)	R^2^	Q_e_ (mg/g)	k_2_ (g mg^−1^min^−1^)	R^2^
OVA	250.50	0.0608	0.797	227.17	0.0003	0.932

**Table 4:** Adsorption dynamic parameters of 3DOM material for adenosine.

	Langmuir Model			Freundlich Model		
	Q_lmax_ (mg/g)	KL (mL/μg)	R^2^	KF(mg/g) (mL/μg)1/n	1/n	R^2^
Adenosine	100.28	0.738	0.984	0.53	0.63	0.975

## Data Availability

The raw data supporting the conclusions of this article will be made available by the authors upon request.

## Supplementary Materials

The following supporting information can be downloaded at: https://www.mdpi.com/article/10.3390/polym16111539/s1, Formulations; Figure S1: TEM and SEM images and size distribution for monodispersed SiO2 particles; Figure S2: SCCB made from monodispersed SiO2 particles of different particle sizes. Reference [54] are cited in the Supplementary Materials.

## References

[1] Soltani S., Jouban A. (2014). Biological sample preparation: Attempts on productivity increasing in bioanalysis. Bioanalysis.

[2] Sun X.Y., Ma R.T., Chen J., Shi Y.P. (2018). Synthesis of magnetic molecularly imprinted nanoparticles with multiple recognition sites for the simultaneous and selective capture of two glycoproteins. J. Mater. Chem. B.

[3] Li D., Li Y., Li X., Bie Z., Pan X., Zhang Q., Liu Z. (2015). A high boronate avidity monolithic capillary for the selective enrichment of trace glycoproteins. J. Chromatogr. A.

[4] Yao J., Wang J., Sun N., Deng C. (2017). One-step functionalization of magnetic nanoparticles with 4-mercaptophenylboronic acid for a highly efficient analysis of N-glycopeptides. Nanoscale.

[5] Gunasekara R.W., Zhao Y. (2017). A General Method for Selective Recognition of Monosaccharides and Oligosaccharides in Water. J. Am. Chem. Soc..

[6] Wang M., Ye F., Wang H., Admassu H., Feng Y., Hua X., Yang R. (2018). Phenylboronic Acid Functionalized Adsorbents for Selective and Reversible Adsorption of Lactulose from Syrup Mixtures. J. Agric. Food Chem..

[7] Kubo T., Furuta H., Naito T., Sano T., Otsuka K. (2017). Selective adsorption of carbohydrates and glycoproteins via molecularly imprinted hydrogels: Application to visible detection by a boronic acid monomer. Chem. Commun..

[8] Wang C., Xu H., Wei Y. (2016). The preparation of high-capacity boronate affinity adsorbents by surface initiated reversible addition fragmentation chain transfer polymerization for the enrichment of ribonucleosides in serum. Anal. Chim. Acta.

[9] Okutucu B., Vurmaz D., Tuncal A., Türkcan C., Aktaş Uygun D., Akgöl S. (2016). Boronate affinity nanoparticles for nucleoside separation. Artif. Cells Nanomed. Biotechnol..

[10] Li D., Chen Y., Liu Z. (2015). Boronate affinity materials for separation and molecular recognition: Structure, properties and applications. Chem. Soc. Rev..

[11] Liu Z., He H. (2017). Synthesis and Applications of Boronate Affinity Materials: From Class Selectivity to Biomimetic Specificity. Acc. Chem. Res..

[12] Siegel D. (2012). Applications of reversible covalent chemistry in analytical sample preparation. Analyst.

[13] Ren L., Liu Z., Liu Y., Dou P., Chen H.Y. (2009). Ring-opening polymerization with synergistic co-monomers: Access to a boronate-functionalized polymeric monolith for the specific capture of cis-diol-containing biomolecules under neutral conditions. Angew. Chem..

[14] Chen M., Lu Y., Ma Q., Guo L., Feng Y.Q. (2009). Boronate affinity monolith for highly selective enrichment of glycopeptides and glycoproteins. Analyst.

[15] Ren L., Liu Z., Dong M., Ye M., Zou H. (2009). Synthesis and characterization of a new boronate affinity monolithic capillary for specific capture of cis-diol-containing compounds. J. Chromatogr. A.

[16] Yang F., Lin Z., He X., Chen L., Zhang Y. (2011). Synthesis and application of a macroporous boronate affinity monolithic column using a metal-organic gel as a porogenic template for the specific capture of glycoproteins. J. Chromatogr. A.

[17] Xue X., Lu R., Liu M., Li Y., Li J., Wang L. (2019). A facile and general approach for the preparation of boronic acid-functionalized magnetic nanoparticles for the selective enrichment of glycoproteins. Analyst.

[18] Gan Q., Lu X., Yuan Y., Qian J., Zhou H., Lu X., Shi J., Liu C. (2011). A magnetic, reversible pH-responsive nanogated ensemble based on Fe3O4 nanoparticles-capped mesoporous silica. Biomaterials.

[19] Süngü Ç., Kip Ç., Tuncel A. (2019). Molecularly imprinted polymeric shell coated monodisperse-porous silica microspheres as a stationary phase for microfluidic boronate affinity chromatography. J. Sep. Sci..

[20] González-Urbina L., Baert K., Kolaric B., Pérez-Moreno J., Clays K. (2012). Linear and nonlinear optical properties of colloidal photonic crystals. Chem. Rev..

[21] Ren X.H., Wang H.Y., Li S., He X.W., Li W.Y., Zhang Y.K. (2021). Preparation of glycan-oriented imprinted polymer coating Gd-doped silicon nanoparticles for targeting cancer Tn antigens and dual-modal cell imaging via boronate-affinity surface imprinting. Talanta.

[22] Senel S., Camli S.T., Tuncel M., Tuncel A. (2002). Nucleotide adsorption–desorption behaviour of boronic acid functionalized uniform-porous particles. J. Chromatogr. B.

[23] Kip Ç., Gülüşür H., Çelik E., Usta D.D., Tuncel A. (2018). Isolation of RNA and beta-NAD by phenylboronic acid functionalized, monodisperse-porous silica microspheres as sorbent in batch and microfluidic boronate affinity systems. Colloids Surf. B Biointerfaces.

[24] Qin X., Zhang Z., Shao H., Zhang R., Chen L., Yang X. (2020). Boronate affinity material-based sensors for recognition and detection of glycoproteins. Analyst.

[25] Sadakane M., Sasaki K., Nakamura H., Yamamoto T., Ninomiya W., Ueda W. (2012). Important property of polymer spheres for the preparation of three-dimensionally ordered macroporous (3DOM) metal oxides by the ethylene glycol method: The glass-transition temperature. Langmuir ACS J. Surf. Colloids.

[26] Liu Q., Feng Y., Huang S., Wu Q., He J. (2015). Preparation of ordered macroporous cinchonine molecularly imprinted polymers and comparative study of their structure and binding properties with traditional bulk molecularly imprinted polymers. Polym. Int..

[27] El-Safty S.A. (2008). Synthesis, characterization and catalytic activity of highly ordered hexagonal and cubic composite monoliths. J. Colloid Interface Sci..

[28] Liang C., Zhang Z., Zhang H., Ye L., He J., Ou J., Wu Q. (2020). Ordered macroporous molecularly imprinted polymers prepared by a surface imprinting method and their applications to the direct extraction of flavonoids from Gingko leaves. Food Chem..

[29] Liu Y.Z., Guo R.T., Duan C.P., Wu G.L., Miao Y.F., Gu J.W., Pan W.G. (2021). Removal of gaseous pollutants by using 3DOM-based catalysts: A review. Chemosphere.

[30] Chen X., Chen Z., Tian R., Yan W., Yao C. (2012). Glucose biosensor based on three-dimensional ordered macroporous self-doped polyaniline/Prussian blue bicomponent film. Anal. Chim. Acta.

[31] Ji X., Li Q., Yu H., Hu X., Luo Y., Li B. (2020). Three-dimensional ordered macroporous ZIF-8 nanoparticle-derived nitrogen-doped hierarchical porous carbons for high-performance lithium-sulfur batteries. RSC Adv..

[32] Fan H.L., Sun T., Zhao Y.P., Shangguan J., Lin J.Y. (2013). Three-dimensionally ordered macroporous iron oxide for removal of H2S at medium temperatures. Environ. Sci. Technol..

[33] Han H., Wang T., Zhang Y., Nurpeissova A., Bakenov Z. (2020). Three-Dimensionally Ordered Macroporous ZnO Framework as Dual-Functional Sulfur Host for High-Efficiency Lithium-Sulfur Batteries. Nanomaterials.

[34] Zhang X., Li L., Wen S., Luo H., Yang C. (2017). Design and synthesis of multistructured three-dimensionally ordered macroporous composite bismuth oxide/zirconia: Photocatalytic degradation and hydrogen production. J. Colloid Interface Sci..

[35] Wang Y., Zhao Q., Han N., Bai L., Li J., Liu J., Che E., Hu L., Zhang Q., Jiang T. (2015). Mesoporous silica nanoparticles in drug delivery and biomedical applications. Nanomed. Nanotechnol. Biol. Med..

[36] Huang Y., Li P., Zhao R., Zhao L., Liu J., Peng S., Fu X., Wang X., Luo R., Wang R. (2022). Silica nanoparticles: Biomedical applications and toxicity. Biomed. Pharmacother..

[37] Guo C., Liu Y., Li Y. (2021). Adverse effects of amorphous silica nanoparticles: Focus on human cardiovascular health. J. Hazard. Mater..

[38] Rimer J.D., Fedeyko J.M., Vlachos D.G., Lobo R.F. (2006). Silica self-assembly and synthesis of microporous and mesoporous silicates. Chemistry.

[39] Thi T.T.H., Cao V.D., Nguyen T.N.Q., Hoang D.T., Ngo V.C., Nguyen D.H. (2019). Functionalized mesoporous silica nanoparticles and biomedical applications. Mater. Sci. Eng. C Mater. Biol. Appl..

[40] Li Z., Barnes J.C., Bosoy A., Stoddart J.F., Zink J.I. (2012). Mesoporous silica nanoparticles in biomedical applications. Chem. Soc. Rev..

[41] Ghimire P.P., Jaroniec M. (2021). Renaissance of Stober method for synthesis of colloidal particles: New developments and opportunities. J. Colloid Interface Sci..

[42] Schedl A.E., Howell I., Watkins J.J., Schmidt H.W. (2020). Gradient Photonic Materials Based on One-Dimensional Polymer Photonic Crystals. Macromol. Rapid Commun..

[43] She H., Ma X., Chang G. (2018). Highly efficient and selective removal of N-heterocyclic aromatic contaminants from liquid fuels in an Ag(I) functionalized metal-organic framework: Contribution of multiple interaction sites. J. Colloid Interface Sci..

[44] Ma X.F., Wu Z.S., Zhang H.Y., Liu Z.Y., Li D.Q. (2016). Mercury Removal by Adsorption on Pectin Extracted from Sugar Beet Pulp: Optimization by Response Surface Methodology. Chem. Eng. Technol. Ind. Chem. Plant Equip. Process Eng. Biotechnol..

[45] Wang M., Ye F., Wang H., Admassu H., Gasmalla M.A., Hua X., Yang R. (2019). High efficiency selective and reversible capture of lactulose using new boronic acid-functionalized porous polymeric monoliths. Chem. Eng. J..

[46] Zhu H., Yao H., Kexu X., Liu J., Yin X., Zhang W., Pan J. (2018). Magnetic nanoparticles combining teamed boronate affinity and surface imprinting for efficient selective recognition of glycoproteins under physiological pH. Chem. Eng. J..

[47] Ye F., Yang R., Hua X., Zhao G. (2014). Adsorption characteristics of rebaudioside A and stevioside on cross-linked poly(styrene-co-divinylbenzene) macroporous resins functionalized with chloromethyl, amino and phenylboronic acid groups. Food Chem..

[48] Fu J., Xin Q., Wu X., Chen Z., Yan Y., Liu S., Wang M., Xu Q. (2016). Selective adsorption and separation of organic dyes from aqueous solution on polydopamine microspheres. J. Colloid Interface Sci..

[49] Hua C., Chen K.M., Guo X.H. (2021). Boronic acid-functionalized spherical polymer brushes for efficient and selective enrichment of glycoproteins. J. Mater. Chem. B.

[50] Su J., He X.W., Chen L.X., Zhang Y.K. (2018). A combination of “thiol-ene” click chemistry and surface initiated atom transfer radical polymerization: Fabrication of boronic acid functionalized magnetic graphene oxide composite for enrichment of glycoproteins. Talanta.

[51] Wang P., Zhu H., Liu J., Ma Y., Yao J., Dai X., Pan J. (2019). Double affinity integrated MIPs nanoparticles for specific separation of glycoproteins: A combination of synergistic multiple bindings and imprinting effect. Chem. Eng. J..

[52] Yao J., Ma Y., Liu J., Liu S., Pan J. (2019). Janus-like boronate affinity magnetic molecularly imprinted nanobottles for specific adsorption and fast separation of luteolin. Chem. Eng. J..

[53] Ektirici S., Göktürk I., Yılmaz F., Denizli A. (2020). Selective Recognition of Nucleosides by Boronate Affinity Organic-Inorganic Hybrid Monolithic Column. J. Chromatogr. B.

[54] Fu J., Chen Z., Wang M., Liu S., Zhang J., Zhang J., Han R., Xu Q. (2015). Adsorption of methylene blue by a high-efficiency adsorbent (polydopamine microspheres): Kinetics, isotherm, thermodynamics and mechanism analysis. Chem. Eng. J..

