# Supplementary material for: Synthesis and Characterization of Boronate Affinity Three-Dimensionally Ordered Macroporous Materials
Source: Polymers (Basel). 2024 May 29;16(11):1539. doi: 10.3390/polym16111539 (PMC11174375; doi:10.3390/polym16111539)
Supplement: Supplementary file 1 [file polymers-16-01539-s001.zip › polymers-2967110-supplementary.pdf]

Synthesis and Characterization of Boronate Affinity Three-Dimensionally Ordered Macroporous Materials

Zhipeng Li †, Luxia Zhang †, Xiangyu Han †, Qinchen An, Mengying Chen, Zichang Song, Linyi Dong, Xianhua Wang * and Yang Yu *

Tianjin Key Laboratory on Technologies Enabling Development of Clinical Therapeutics and Diagnostics, School of Pharmacy, Tianjin Medical University, Tianjin 300070, China; lizhipeng152226@163.com (Z.L.); m15735177825_1@163.com (L.Z.); handream@foxmail.com (X.H.); aqc2001@163.com (Q.A.); chenmengying1999@163.com (M.C.); szc380606848@163.com (Z.S.); donglinyi@tmu.edu.cn (L.D.)

* Correspondence: xianhua.w@163.com (X.W.); yuyang@tmu.edu.cn (Y.Y.)

† These authors contributed equally to this work.

S 1. Experimental section

1.1 Adsorption dynamics

The data was fitted using Langmuir model (1) and Freundlich model (2).

$$Q_e = \frac{Q_{lmax} K_L C_e}{1+K_L C_e} \quad (1)$$

$$Q_e = K_F C_e^{1/n} \quad (2)$$

In which, Q_e stands for the absorption quantity at equilibrium and Ce is the final concentration of absorbate in the sample solution at equilibrium. In formula (1), Q_{lmax} is theoretical maximum absorption capacity and K_L represents the affinity constants in Langmuir model. In formula (2), n is correlated with absorption driving force. The value of 1/n indicates whether absorption is easy (0.1<1/n<0.5), difficult (0.5<1/n<1) or very difficult (1/n>1). K_F is the absorption equilibrium constant in Freundlich model.

The thermodynamic parameters, including Gibbs free energy change (G^0), enthalpy change (H^0) and entropy change (S^0) [1], was used to describe thermodynamic

behavior of OVA absorption by 3DOM and was calculated using following formulas (3) and (4):

$$\Delta G^0 = -RT\ln(M/K_L) \quad (3)$$

$$\ln(M/K_L) = -\Delta H^0/(R\times T) + \Delta S^0/R \quad (4)$$

The enthalpy change (H^0) and entropy change (S^0) were calculated according to Van't Hoff equation (4), in which, M (g/mol) is the molecular weight of OVA (46 Kda), R is the universal gas constant [8.314 J/ (mol·K)] and K is the system temperature. K_L is the equilibrium constant of Langmuir model.

1.2 Absorption kinetics

Pseudo first-order kinetic equation (5) and pseudo second-order kinetic equation (6) were applied to describe the kinetic absorption process:

$$Q_t = Q_e - Q_e e^{-k_1 t} \quad (5)$$

$$Q_t = \frac{k_2\,Q_e^2\,t}{1+k_2 Q_e t} \quad (6)$$

Where, Q_t denotes the absorption quantity at the time t, and Q_e denotes the absorption quantity at equilibrium. In (5), k_1 is the speed constant in pseudo first-order kinetic model; in (6), k_2 is the speed constant in pseudo second-order kinetic model.

S 2. Figures

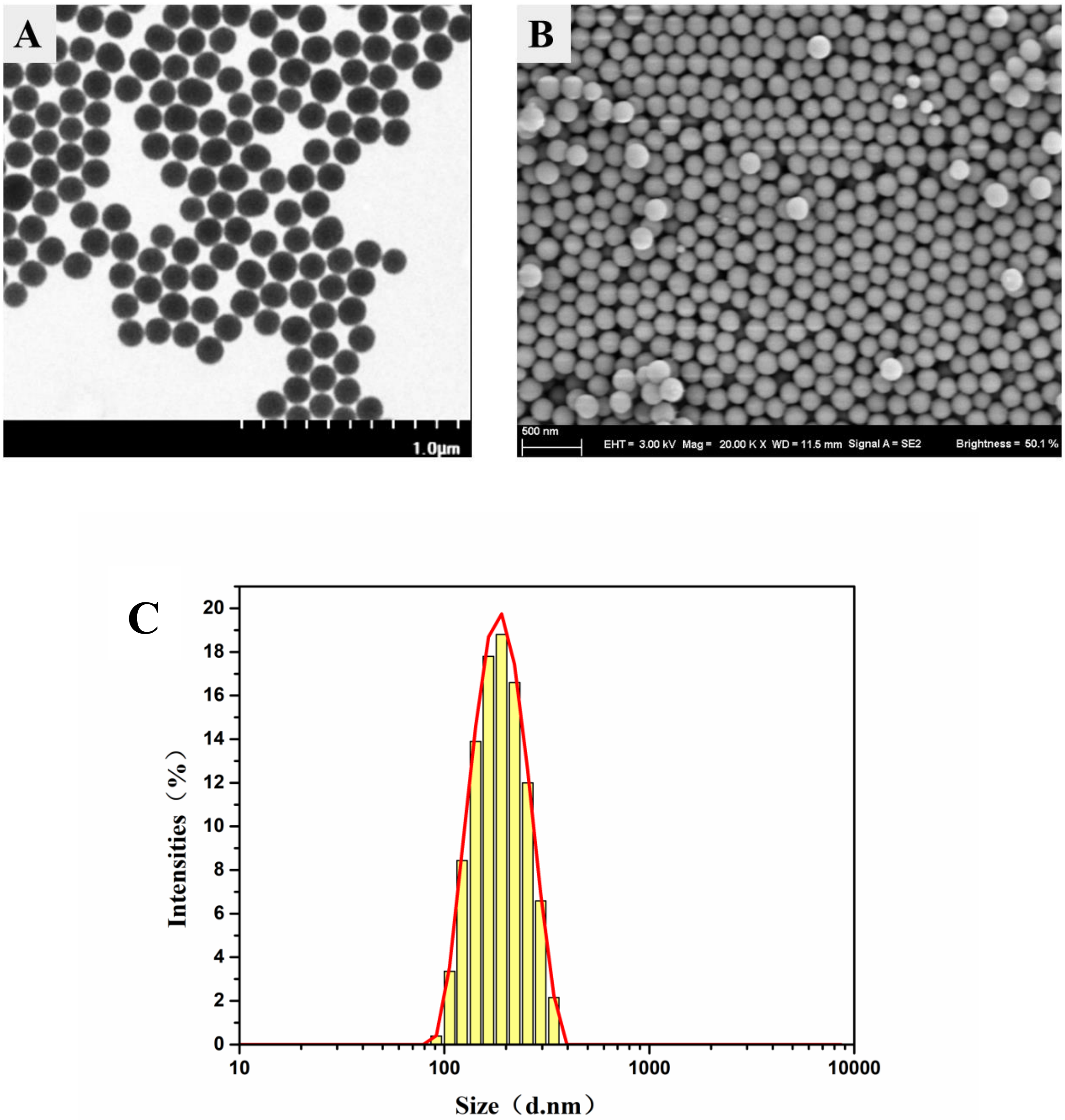

Figure S1. TEM (A) and SEM (B) images for monodispersed SiO_2 particles. (C) The size distribution of monodispersed SiO_2 particles.

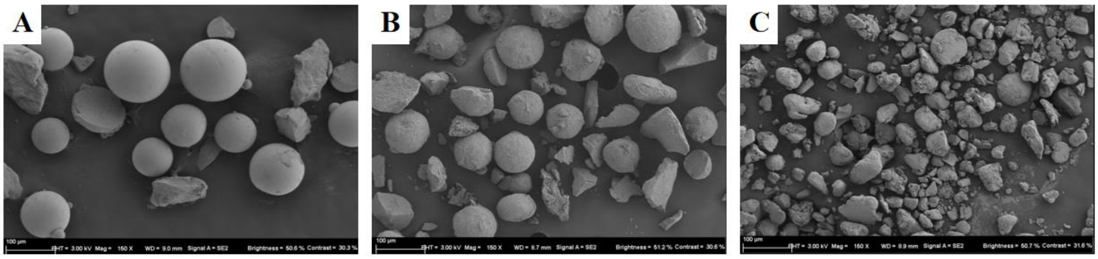

Figure S2. SCCB made from monodispersed SiO_2 particles of different particle sizes. (A) 170 nm; (B) 200 nm; (C) 250 nm.

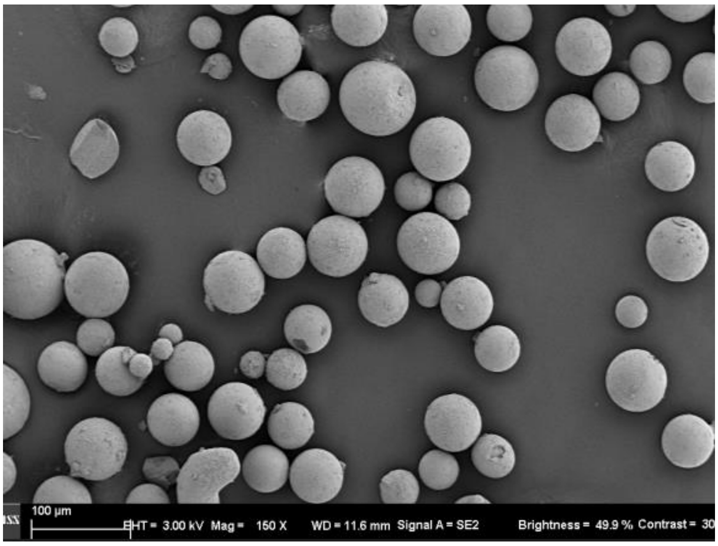

Figure S3. SCCB with lower magnification (150×)

References:

[1] J. Fu, Z. Chen, M. Wang, S. Liu, J. Zhang, J. Zhang, R. Han, Q. Xu, Adsorption of methylene blue by a high-efficiency adsorbent (polydopamine microspheres): Kinetics, isotherm, thermodynamics and mechanism analysis, Chemical Engineering Journal, 259 (2015) 53-61.
